# Using a Revised Protein-Sparing Modified Fast (rPSMF) for Children and Adolescents with Severe Obesity: A Pilot Study

**DOI:** 10.3390/ijerph16173061

**Published:** 2019-08-23

**Authors:** Ihuoma Eneli, Jinyu Xu, Alexis Tindall, Rosanna Watowicz, Jennifer Worthington, Kelly Tanner, Keeley Pratt, Marnie Walston

**Affiliations:** 1 Center for Healthy Weight and Nutrition, Nationwide Children’s Hospital, Columbus, OH 43205, USA; 2 Department of Pediatrics, The Ohio State University, Columbus, OH 43210, USA; 3 Department of Nutrition, Case Western University, Cleveland, OH 44106, USA; 4 Division of Clinical Therapies, Nationwide Children’s Hospital, Columbus, OH 43205, USA; 5 Department of Human Sciences, The Ohio State University, Columbus, OH 43210, USA; 6 Department of Pediatrics, Akron Children’s Hospital, Akron, OH 44308, USA

**Keywords:** severe obesity, protein-sparing modified Fast, children and adolescents, weight management

## Abstract

Treatment options are limited for children and adolescents with severe obesity. One alternative treatment is the protein-sparing modified fast (PSMF), a low-carbohydrate, high-protein diet that can result in substantial weight loss. The aim of the study is to evaluate the adherence and efficacy of a revised PSMF (rPSMF) for severe obesity in a pediatric tertiary care weight-management program. The rPSMF with 1200–1800 calories, 40–60 g of carbohydrate/day and 1.2–1.5 g protein/kg of ideal bodyweight was implemented over 12 months. Twenty-one participants enrolled in the study. Mean age 16.2 ± 1.4 years, females (76.2%) and mean weight at baseline was 119 ± 19.9 kg. Regardless of adherence to the rPSMF, the mean weight change at 1 month was −3.7 ± 3.5 kg, (range −13.5 kg to 0.9 kg); at 3 months was −5.5 ± 5.1 kg, (range −19.3 kg to 1.8 kg) and at 6 months was −4.7 ± 6.6 kg, (range −18.3 kg to 8.6 kg). At 12 months, the mean weight change was −1.3 ± 10.6 kg (range −17.7 kg to 14.8 kg). Parent and child-reported physical and psychosocial quality of life (HRQOL) improved. Despite limited adherence, the rPSMF diet resulted in clinically significant weight loss and improved HRQOL for children and adolescents with severe obesity.

## 1. Introduction

Childhood obesity is a significant public health problem. There has been a concerted effort to identify treatment strategies that are effective, especially for the subset of youth with severe obesity. Severe obesity is defined as a body mass index (BMI) greater than 120% of the absolute BMI at the age and sex-specific 95th percentile. This cut-off criterion is analogous to an adult with a BMI ≥ 35 [1]. The consequences of severe childhood obesity can be devastating, as this disease tracks into adulthood [2]. Children with severe obesity are also at increased risk for serious comorbidities such as hypertension, type 2 diabetes, and premature death [3].

In 2013, the American Heart Association highlighted a gap in treatment for more than 5 million children in the United States with severe obesity and called for alternative approaches to weight management [3]. Conventional treatment for severe obesity within pediatric weight management centers often involves nutrition and physical activity lifestyle interventions delivered by a multidisciplinary team [4,5,6]. Unfortunately, these interventions are often poorly effective for this subpopulation of children [7,8,9], while bariatric surgery, an effective intervention for severe obesity, is not readily accessible [10]. The 2007 Expert Committee Recommendations for the Prevention, Assessment, and Treatment of Childhood Obesity recommends the use of medically supervised low-calorie diets for children with severe obesity in addition to bariatric surgery [11]. An example is the protein-sparing modified fast (PSMF), an extremely low-carbohydrate, high-protein, reduced-calorie diet. With the PSMF, the protein component of the meal plan is “spared” (i.e., maintained at normal or high levels in the diet) which helps to increase satiety and suppress appetite while preserving lean body mass. In the absence of carbohydrates, increased lipolysis occurs leading to fat breakdown and weight loss. The process of lipolysis and fatty acid oxidation produces ketones, which further suppresses the appetite [12].

Extant literature on the PSMF in both adults and adolescents is encouraging [9,13,14,15,16,17,18,19,20], yet studies on the use of the PSMF in children and adolescents to treat obesity are limited. In a study of adolescents with severe obesity who previously failed at conventional lifestyle modification, the PSMF produced a clinically significant mean decrease of 9.8% of body weight over 6 months [15]. A case series using a revised PSMF protocol with carbohydrate intake limited to 20 g per day in both an inpatient and outpatient setting reported an average weight loss of 1–2 pounds a week and improvement in comorbidities [19]. Traditionally, the PSMF is categorized as a very low-calorie diet (VLCD), with significant calorie restriction, sometimes as low as 500–800 kcal per day [20,21]. However, VLCDs (when provided mostly as liquid meal replacements) have raised safety concerns in the past [18,22]; so recent studies using the PSMF have been less stringent with caloric restriction and have demonstrated improved safety profiles [15,16,19].

Most of the studies in the pediatric population have been hampered by small sample sizes, follow up periods of 6 months or less and low retention rates [15,17,23]. In addition, not much is known about the relationship between varying levels of adherence with the diet and weight-related outcomes or quality of life. This paper will describe the outcomes of a 12-month pilot study to examine the adherence and efficacy of a revised PSMF (rPSMF) with a more liberal energy intake than a traditional PSMF, for children and adolescents with severe obesity in a multidisciplinary tertiary care pediatric weight-management clinic.

## 2. Method

### 2.1. Study Design and Participants

This was a 12-month prospective cohort study in a clinical setting for children and adolescents between 11–18 years. The study protocol is described in depth elsewhere [24]. Eligibility criteria included: (1) presence of severe obesity categorized as Class 2 (defined as a BMI ≥ 120% of the 95th age and sex-specific percentile, or a BMI ≥ 35) or Class 3 (defined as a BMI ≥ 140% of the 95th age and sex-specific percentile, or a BMI ≥ 40) [1]; (2) pubertal Tanner stage 3 or above to address concerns about linear growth; and (3) the presence of any obesity-related comorbidities. For participants between 11–13 years, at least one severe comorbidity (e.g., obstructive sleep apnea, type 2 diabetes, non-alcohol fatty liver disease, slipped capital femoral epiphysis, Blount disease, or idiopathic intracranial hypertension (pseudotumor cerebri)) had to be present. In addition, participants had to have adequate social support and a stable mental health status. Exclusion criteria were (1) a history or presence of arrhythmia; (2) impaired renal function defined as creatinine >0.9 mg/dL or GFR < 90 mL/min/1.73 m^2^; (3) elevated baseline uric acid; (4) a positive pregnancy test; and (5) lack of medical insurance. If the participant met the eligibility criteria, the rPSMF would be offered as a treatment option. If the participant and custodial parent/caregiver agreed to use the rPSMF, they were approached to enroll in the study. The study was approved by the Hospital Institutional Review Board (IRB15-00667) and listed with ClinicalTrials.gov Identifier NCT03899311. All participants and a parent or caregiver completed a written informed consent or assent.

### 2.2. Revised Protein Sparing Modified Fast (rPSMF)

**Figure 1:**
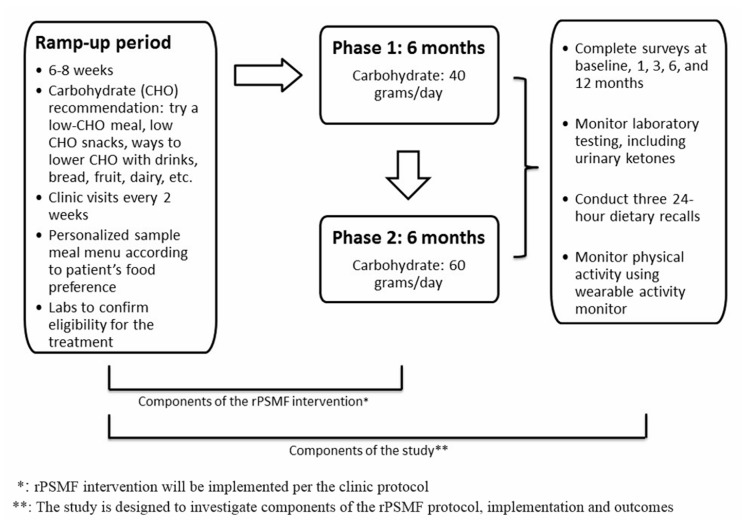
Schematic representation of the rPSMF intervention and study protocol.

The traditional PSMF diet was revised for use in this pilot study to allow for more liberal caloric intake (1200–1800 kcal) compared to the typical very low calorie diet (600–1000 kcal). Henceforth, it will be referred to as the revised PSMF (rPSMF). The rPSMF is described in depth elsewhere and is briefly summarized here (Figure 1 (Reprinted from [24] Figure 1, Copyright (2019), with permission from Elsevier.)). The intervention was provided in three phases. Phase 1 was the most restrictive, allowing for 40 g of carbohydrate per day for 6 months. During Phase 2, daily carbohydrate intake was increased to 60 g per day for 6 months through the introduction of fruits and low-fat dairy products. Finally, in Phase 3 (at 12 months), daily carbohydrate intake was gradually increased to an eventual set-point of approximately 100–200 g of carbohydrate per day and a desired caloric intake that did not exceed 2000 calories per day. Daily protein intake was 1.2–1.5 g protein per kilogram of ideal bodyweight, with an emphasis on low fat and lean protein options. Meal replacement drinks or supplements were not specifically recommended or prohibited. Calorie intake ranged from 1200–1800 calories/day and was based on child’s age, estimated ideal body weight, and level of physical activity. Each participant worked with a dietitian over several days on meal plans using a food preference and shopping list. A daily intake of 2–3 L of fluid was recommended, and a daily multivitamin, calcium supplement, and vitamin D supplement were prescribed for the duration of the intervention.

Laboratory tests were conducted to confirm eligibility for rPSMF and to monitor for comorbidities such as type 2 diabetes (blood glucose, glycosylated hemoglobin), non-alcoholic fatty liver disease (alanine transaminase), dyslipidemia (triglycerides, total cholesterol, LDL, HDL), and nutritional status (calcium, 25-OH Vitamin D, Vitamin B1 (Thiamine), B6 (Pyridoxine), B12 (Cyanocobalamin) and Folic acid).

Each participant had at minimum a 6–8 week “ramp-up” phase during which a trial run of components of the rPSMF was conducted. For instance, participants were asked to discontinue sugar-sweetened beverages, to try a low-carbohydrate snack and/or dinner options from a meal plan, and/or increase the amount and variety of vegetables they consumed. If the participant was unable to achieve the goals over the 6–8 weeks, the ramp up stage was extended or an alternate treatment was recommended.

Participants outlined their activity goals and plan with a physical therapist [24]. Participants were provided with a FitBit [25] to wear throughout the study and were assigned incremental daily step goals (ranging from 2500 to 12,500 steps) based on their activity level at baseline. New step goals were given when a goal was achieved for 5 consecutive days.

### 2.3. Data Collection, Retention and Adherence

Data collection was standardized to align with the rPSMF clinical protocol [24]. The participant was enrolled in the study following successful completion of the ramp-up period. Surveys were completed at baseline, 1, 3, 6, and 12 months following initiation of the rPSMF. The survey included questions about demographic information, diet and physical activity, risk factors, and family medical history related to obesity [24]. Adherence with the rPSMF was determined by the registered dietitians (RDs) based on 24-h dietary recalls and food frequency questionnaires documented at each clinic visit. If adolescents consumed 40 ± 10 g of carbohydrates per day over the past month, adolescents were considered “adherent”. If the adolescent noted attempts to consume low-carbohydrate meals and/or snacks throughout the past month, but did not always reach the goal of 40 ± 10 g of carbohydrates per day, they were considered “moderately adherent”. If the adolescent reported none or very few attempts to consume low-carbohydrate meals and/or snacks within the past month, they were considered “not adherent”. For the analysis, adherence was categorized as a dichotomous variable into “not adherent” or “adherent” by combining the moderately adherent and adherent categories. Participants were advised to obtain daily urinalysis for ketone testing for the first month. While the presence of ketosis can be used as a measure of adherence, for this protocol it was employed to ensure safety of the intervention and avoid severe ketosis.

Adherence rate to the rPSMF was defined as percent of participants who were noted “adherent” on dietitian evaluation during the assessment clinic visit. If a participant was still enrolled in the study but did not attend their clinic visit, they were classified as non-adherent as there were no dietitian notes for evaluation, thus adopting a “worst-case” scenario. Retention rate was calculated as the percent of participants who completed the study surveys at 1, 3, 6, and 12 months.

### 2.4. Anthropometrics

Trained clinic staff obtained measures of weight to the nearest 0.1 kg using a Tronix digital scale with the child wearing only light clothing, and height without footwear to the nearest 0.1 cm with a digital stadiometer (SECA Model 2641900009). Body mass index (BMI) was calculated and percent change in BMI and BMI z-score were derived using the 2000 Center for Disease Control and Prevention (CDC) age- and sex-specific references [26]. The percentage of the 95th percentile for BMI (%BMIp95), defined as the ratio of the BMI to the age and sex-specific BMI at the 95th percentile multiplied by 100, was calculated. The %BMIp95 is a more stable measure for severe pediatric obesity [27].

### 2.5. Health-Related Quality of Life

Health-related quality of life (HRQOL) was assessed using the 23-item Pediatric Quality of Life (PedsQL) 4.0 inventory, a validated measure used extensively to assess children with pediatric chronic health conditions including obesity [28]. The child and caregiver/parental versions of the PedsQL4.0 were completed by the participant and their parent, respectively. The PedsQL4.0 has four subscales: physical (eight items), emotional (five items), social (five items), and school function (five items). Each item has five choices on a Likert scale: 0 = never a problem, 1 = almost never a problem, 2 = sometimes a problem, 3 = often a problem, and 4 = almost always a problem. Raw scores are reversely scored and linearly transformed to a 0–100 scale. The PedsQL4.0 score is calculated using the average of the 23 items for the total score and the averages of the items for each subscale. A psychosocial subscale score is the average of the emotional, social, and school subscales. Higher scores indicate better HRQOL.

### 2.6. Data Analysis

Descriptive statistics were used to calculate participants’ demographic characteristics. Change in weight, BMI, %BMIp95, and BMI z-score were calculated at 1, 3, 6, and 12 months. In the analysis, weight loss was used as a dichotomous variable, adolescents either lost weight or gained weight/stayed the same weight. Due to the non-normal distribution of child weight measurements, non-parametric tests (Wilcoxon) were used to compare adherent and non-adherent groups at 1, 3, 6, and 12 months. Repeated measures analysis of variance were used to examine anthropometric changes over time.

## 3. Results

Over a 6-month period, 65 participants in the tertiary care pediatric weight management clinic met the eligibility criteria and were offered the rPSMF as a treatment option. Twenty-nine (44.6%) participants moved ahead with the ramp-up phase. Twenty-one participants successfully completed the ramp up phase, started the rPSMF, and enrolled in the study. The demographic and clinical characteristics of the participants who were eligible and offered the rPSMF as an option and participants who enrolled in the study are shown in Table 1. Eight participants attempted the rPSMF ramp up phase, but did not continue with the intervention. They had a mean age of 16 years, 25% were male, 50% identified as Non-Hispanic White, and 37.5% had at least one serious weight-related comorbidity.

### 3.1. Study Retention and Adherence to the rPSMF

The combined retention rate for participants and parent/caregivers at 1 month, 3 months, 6 months, and 12 months was 95%, 90%, 86%, and 81%, respectively. For the adolescents, completed survey data was available for analysis for 21 (100%) adolescents at baseline, 20 (95.2%) participants at 1 month, 19 (81.0%) at 3 months, 18 (71.4%) at 6 months, and 17 (61.9%) at 12 months. For the parents, 20 parents (100%) provided data at baseline, 18 (90%) at 1 month, 17 (85%) at month 3, 17 (85%) at month 6, and 17 (85%) at month 12.

**Figure 2:**
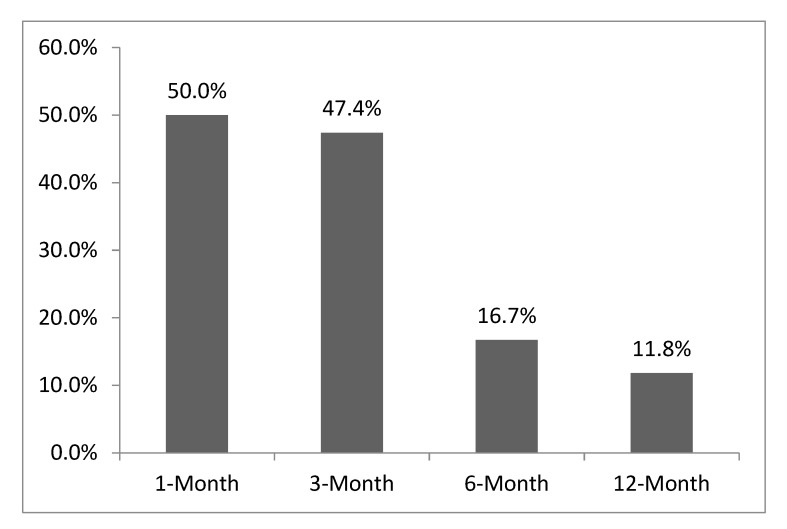
rPSMF adherence rate over 12 months.

Adherence rate to the rPSMF was approximately 47.4% and 16.7% at 3 and 6 months, respectively (Figure 2). When participants who did not attend their clinic visits or had dropped out of the study were excluded, adherence rate was 58.3% and 37.5% at 3 and 6 months, respectively. The pattern of adherence to the rPSMF varied for each participant. None of the adolescents were fully adherent at all time points of the study. Of the participants who were adherent at 1 month (n = 10), only seven remained adherent at 3 months, and only two remained adherent at 6 months. Two participants who had not been adherent at 1 month, were however adherent at 3 months.

### 3.2. Anthropometrics

Regardless of adherence to the rPSMF, there was a decrease in all weight-related measures (Table 2). The largest decline in weight-related measures occurred at 3 and 6 months. The mean weight change at 1 month was −3.7 ± 3.5 kg (range −13.5 kg to 0.9 kg) and at 3 months was −5.5 ± 5.1 kg, (range −19.3 kg to 1.8 kg). At 12 months, the mean weight change was −1.3 ± 10.6 kg, (range −17.7 kg to 14.8 kg).

**Figure 3:**
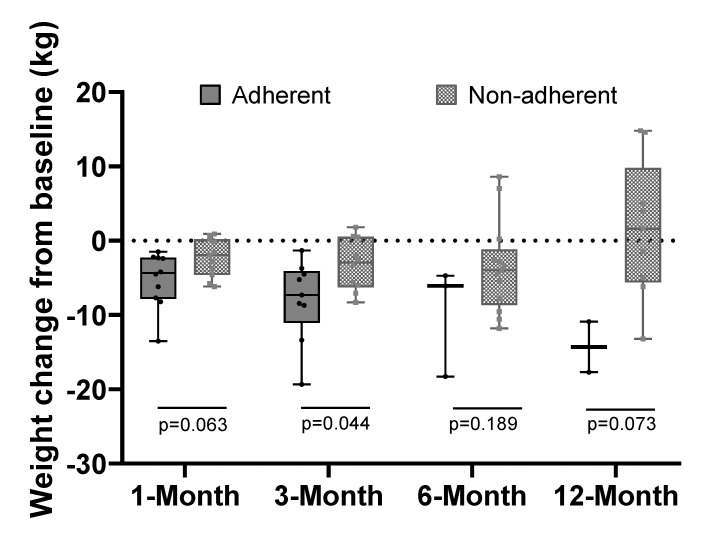
Weight change by adherence to rPSMF.

At 3 months, 9 participants who were adherent to rPSMF lost significantly more weight than the 10 participants who were not adherent (−8.0 ± 5.5 kg vs. −3.2 ± 3.5 kg, *p* = 0.04). The results were similar at 1, 6 and 12 months with a trend towards significance at 1 and 12 months (Figure 3). Despite the high rates of non-adherence, 11 (52.4%) and 8 (38.1%) participants lost ≥5% and ≥7% of their body weight, respectively at some point in study period. At 3 months, seven participants (33%) had lost ≥5% of their body weight. Of these participants, five were able to sustain this level of weight loss at 6 and 12 months. Compared to their baseline weight, five participants had lost ≥5% and three had lost ≥7% in body weight at 12 months.

### 3.3. Physical and Psychosocial HRQOL

**Figure 4:**
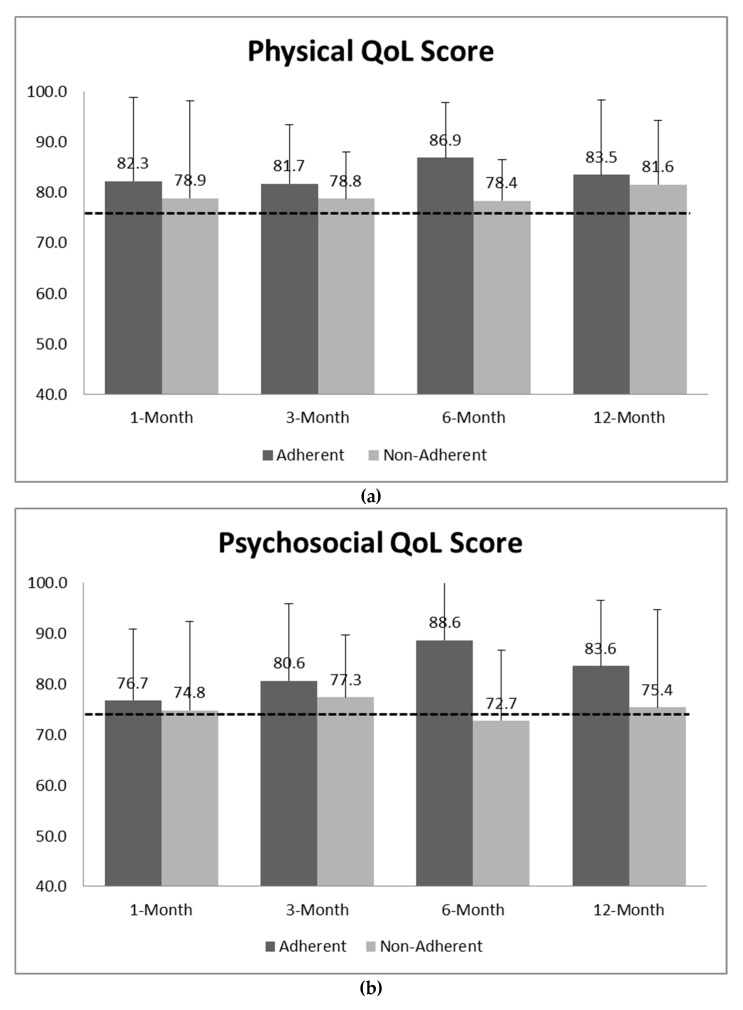
Participant-reported physical (**a**) and psychosocial (**b**) HRQOL by adherence (Dotted lines: Mean score at baseline).

Mean physical and psychosocial HRQOL subscale scores improved from baseline regardless of adherence status. At baseline, the average score of physical and psychosocial subscales reported by participants was 74.9 ± 17.3 and 74.7 ± 15.5, respectively. The scores increased over the 12 months for physical (82.4 ± 13.3) and psychosocial (79.0 ± 16.9) subscales. Participants who were adherent reported higher physical and psychosocial scores at each time point compared to their non-adherent peers (Figure 4). The average psychosocial score for adherent adolescents at 12 months improved by 12% from baseline and the physical health score improved by 11% (Figure 4) from baseline among adherent participants. Although the total PedQL4.0 and subscale scores reported by parents were lower compared to their children’s scores, they increased in a similar fashion across the study (Data not shown).

### 3.4. Laboratory Studies

All participants had baseline laboratory tests. Only 70% (*n* = 14) had follow up laboratory results at 6 and/or 12 months. Participants who did not attend their clinic visits were less likely to have laboratory studies at 6 or at 12 months. At baseline, all the subjects had normal vitamin profiles (B1, B6, B12, Folic acid) and insufficient vitamin D (10–34 ng/mL); while six (28.6%) had elevated cholesterol (≥200 mg/dL), six (28.6%) had borderline elevated cholesterol (170–199 mg/dL) but only three (14.3%) had elevated glycosylated hemoglobin between 5.7–6.4 mg/dL. Of those participants with abnormal values at baseline, eight (83%) and two (66.7%) improved their cholesterol and glycosylated hemoglobin levels, respectively. Of the subjects with normal or borderline cholesterol, none became abnormal. Vitamin D levels remained insufficient at 6 and 12 months. Two of the three participants with abnormal ALT at baseline (≥40 U/L) had improved levels at 6 and 12 months. In three participants with normal ALT level at baseline, their level was abnormal 1 month post initiation of the rPSMF, but normalized at 3 months.

## 4. Discussion

This pilot study is foundational for the emerging literature on the use of specialized diets in clinical settings for children and adolescents with severe obesity. Since the use of specialized diets for pediatric obesity is not widespread, we assessed the effectiveness of the rPSMF for weight loss among adolescent and the impacts on HRQOL. As it is often challenging to follow a restrictive diet, we examined the relationship between adherence with the rPSMF and weight-related changes. Overall, there was low adherence to the recommended rPSMF carbohydrate and calorie recommendations throughout the study. Yet, despite this poor adherence to the rPSMF as prescribed, participants experienced significant weight loss and both children/adolescent and parent perceptions of HRQOL improved. These paradoxical results raise questions about the etiology for the weight loss observed in the study. It is possible that only specific components of the intervention may be beneficial (e.g., the structured nature of the recommendations). It may be that providing a more structured weight loss plan around a specific concept, in this case, decreasing carbohydrates while increasing protein, may make families more aware of their food choices and food quality. In doing so, they become selective, favoring food choices with lower caloric density and consuming fewer daily calories than their typical diet.

Alternatively, it may be that the diet choices in the rPSMF, which emphasized options such as water or diet drinks, vegetables, lean meats and fish, led to a decrease in intake of typical processed foods found in American diets. Hall et al. reported in a recent study that when adults were offered diets rich with processed foods, their caloric intake increased by an average of 500 calories more than when they were offered a diet with minimally processed foods, such as whole grains, fresh fruits and vegetables, lean proteins, nuts, and seeds [29]. It is also possible that the weight loss occurred due to the increased engagement with the multidisciplinary team or potential increase in activity related to the use of wearable technology. Further testing of potential components of the rPSMF (e.g., food choices, confidence, satisfaction, contact hours, level of support from family and peers) with the addition of a control group is a reasonable next step in exploring the relationship between the use of rPSMF, adherence and weight loss.

The largest declines in weight loss were at 3 and 6 months, with some weight regain occurring by 12 months. This pattern of weight loss is similar to other PSMF studies in adolescents [15,16]. Interestingly, the recommended daily carbohydrate intake was increased from 40 to 60 g at 6 months. Periods of transition for a low carbohydrate diet may make adherence to the diet more difficult, especially as the variety of foods expands to include options like fruits and dairy. Despite the limited adherence, the magnitude of weight loss was clinically significant (Table 2). More than half of the participants lost ≥5% of their body weight at some point in 12 months. Losing 5% or more of your body weight (also the equivalent to a BMI z-score decline of 0.15–0.20) is correlated with improved cardiac and metabolic outcomes [11]. Further, there was a great deal of individual variability in the amount of weight lost. It is possible that the rPSMF may be more effective for certain patients, a speculation that needs to be further explored.

The adherence to the rPSMF and attendance at corresponding clinic visits dropped through the study period. High dropout or attrition rates are, unfortunately, common among pediatric weight management interventions, with reported rates as high as 25–80% [30,31]. In addition, children and adolescents with severe obesity tend to do poorly with adherence in traditional pediatric weight management interventions [8,30,32]. In a clinic-based study of the PSMF for adolescents, the dropout rate was as high as 50% across 12 months [15]. Even with the challenges with retention and adherence, the children/adolescents in this study also experienced significant improvements in weight loss. Pratt et al. in a mixed methods study of the rPSMF with this study population illuminated some of the reasons underlying this finding [33]. Most adolescents and parents reported the outcome they liked the most was “weight loss”. Compared with traditional lifestyle intervention, weight loss was greater and more rapid [3,9]. Other positive aspects of the rPSMF reported by parents included “buying low carb (ohydrate) foods and limiting low carb (ohydrate) options in the home” for the whole family, making healthier nutrition choices” by reading food labels, limiting portion size and avoiding energy-dense foods [33].

Similar to weight loss, participants experienced positive increases in HRQOL, specifically psychosocial and physical health scores, across the study regardless of adherence to the rPSMF. Prior research has documented associations between severe obesity in children/adolescents and impaired HRQOL [34,35,36] and that HRQOL improves over the course of pediatric weight management interventions with or without weight loss [37]. Participants who were adherent had greater increases in HRQOL at 6 and 12 months. It is possible that adolescents who are adherent may feel accomplished, receive positive feedback from their healthcare providers and/or additional support from their parents, which in turn positively enhances their HRQOL. This assumption merits further study as children or adolescents who feel their HRQOL is “good” or improving may be more likely to stick with the rPSMF or components of the rPSMF in the long-term, despite the exacting nature of the diet. The rPSMF did not lead to vitamin deficiencies or significant worsening of comorbidities. The transient elevation in liver function tests that occurred within the first month of instating the rPSMF in three participants may have been due to sludging in the gall bladder, which is often seen when rapid weight loss occurs over a short period of time.

A strength of the study is the rPSMF protocol. Outlined in further detail elsewhere, the protocol is evidence-based and was developed to be pragmatic and reproducible in any tertiary care pediatric obesity program [24]. With a daily intake of 1200–1800 calories, the rPSMF protocol takes into consideration the basal metabolic needs of the child or adolescent and growth, and thus, the caloric restriction was not excessive. In addition, processes to monitor for safety such as regular clinic visits and laboratory tests were incorporated.

This pilot study has limitations. As this is a cohort study implemented within a typical tertiary care obesity program setting, selection bias must be considered. The clinic team may have offered the rPSMF to families they felt were more likely to be successful with the rigor expected of the intervention. The study is also encumbered by the fact that adherence to the diet was obtained primarily through self-report. While home ketone testing was obtained for the first month, its use was non-consistent and thus could not be reliably reported as a measure of adherence. Interestingly, adherence was very low in spite of the use of self-report, suggesting that the social desirability often seen with dietary self-report may not have occurred in any appreciable manner in the study. As a pilot study, the sample size was small; however, it is still larger than recent published studies on the PSMF especially in adolescents [15,19,23]. Despite these limitations, this study provides incremental knowledge about the pragmatic use of the PSMF in a pediatric population. This study addressed whether strict adherence to a low-carbohydrate diet is absolutely necessary in order to see beneficial results, raising a number of mechanistic hypotheses e.g., the relationship between adherence, HRQOL and weight loss that merits further study.

## 5. Conclusions

Despite limited adherence, the rPSMF resulted in clinically significant weight loss and improvements in HRQOL. With rates of severe obesity among children and adolescents and limited effective interventions, the PSMF offers a viable alternate treatment option especially when rapid and short-term weight loss in the presence of serious comorbidities is needed or desired as a bridge to other treatments such as bariatric surgery.

## Figures and Tables

**Table 1:** Demographic and clinical characteristics of the rPSMF eligible participants.

	Participants *N* = 21 (%)	Non-Participants *N* = 44 (%)	*p* Value
Age in years (Mean ± SD)	16.1 ± 1.5	15.9 ± 1.7	0.42
Sex (Female)	16 (76.2)	25 (56.8)	0.13
Race			0.80
African American	9 (42.8)	19 (43.3)	
Hispanic	1 (4.8)	2 (4.5)	
Non-Hispanic White	11 (52.4)	21 (47.7)	
Other	0	2 (4.5)	
Obstructive Sleep Apnea	2 (9.5)	7 (15.9)	0.49
Type 2 Diabetes	0	3 (6.8)	0.22
Dyslipidemia	9 (42.9)	13 (29.6)	0.29
Non-alcoholic fatty liver disease	1 (4.8)	9 (20.5)	0.10
Insurance type			0.87
Medicaid	11 (52.4)	24 (54.6)	
Private	10 (47.6)	20 (45.4)	
Weight, kg (Mean ± SD)	119.3 ± 19.9	119.8 ± 21.7	0.70
BMI (Mean ± SD)	41.9 ± 6.2	42.2 ± 6.5	0.82
%BMIp95 (Mean ± SD)	146.9 ± 21.8	149.8 ± 21.7	0.73
BMI z-score (Mean ± SD)	2.50 ± 0.27	2.58 ± 0.25	0.90

**Table 2:** Change in weight, BMI, BMI z-score and %BMIp95 with the rPSMF over 12 months.

		Baseline	Change from Baseline			
			1 Month	3 Months	6 Months	12 Months
Weight (kg)	Mean (SD)	119.9 (20.3)	−3.7 (3.5)	−5.5 (5.1)	−4.7 (6.6)	−1.3 (10.6)
	*p* value		0.72	0.005 **	0.04 **	0.35
BMI	Mean (SD)	42.0 (6.2)	−1.3 (1.2)	−1.9 (1.9)	−2.1 (2.3)	−0.9 (3.2)
	*p* value		0.94	0.006 **	0.05 **	0.23
Percent BMI	Mean (SD)	100	−3.3 (3.2)	−4.6 (5.1)	-5.3 (5.7)	−2.2 (8.4)
	*p* value		0.28	0.02 **	0.38	0.71
BMI z score	Mean (SD)	2.50 (0.27)	−0.07 (0.08)	−0.11 (0.13)	−0.15 (0.14)	−0.13 (0.21)
	*p* value		*0.35*	0.01 **	*0.15*	*0.73*
%BMIp95	Mean (SD)	146.7 (22.1)	−5.1 (4.2)	−7.4 (6.8)	−9.2 (7.6)	−6.2 (10.8)
	*p* value		0.43	0.006 **	0.06	0.18

**: repeated measures analysis of variance, *p* < 0.05.
